# Case study of a critically ill person with COVID-19 on ECMO successfully treated with leronlimab

**DOI:** 10.1016/j.jtauto.2021.100097

**Published:** 2021-03-23

**Authors:** Sohier Elneil, Jacob P. Lalezari, Nader Z. Pourhassan

**Affiliations:** a University College London, National Hospital for Neurology and Neurosurgery, London, UK; b Quest Clinical Research, 2300 Sutter Street, Suite #202 & 208, San Francisco, CA, 94115, USA; c CytoDyn, 1111 Main Street, Suite 660, Vancouver, WA, 98660, USA

**Keywords:** Leronlimab, Coronavirus-disease-2019-(COVID-19), SARS-CoV-2, Extracorporeal-membrane-oxygenation-(ECMO)

## Abstract

The number of confirmed cases of infection with SARS-CoV-2, the virus causing Coronavirus disease 2019 (COVID-19), continues to increase and is associated with substantial morbidity and mortality in virtually every country in the world. Although in the long-term mass vaccinations remains the most promising approach to control the pandemic, evidence suggests that new variants of the virus have emerged that may be able to evade the immune responses triggered by current vaccines. Therefore despite the recent approval of a number of SARS-CoV-2 vaccines there remains considerable urgency for effective treatments for COVID-19. Severe-to-critical COVID-19 has been shown to be associated with a dysregulated host immune response to SARS-CoV-2 with elevated levels of C–C chemokine receptor type 5 (CCR5) ligands including chemokine C–C ligands 3, 4, 5, as well as interleukins 6 and 10. Leronlimab, a CCR5-specific humanised IgG4 monoclonal antibody originally developed for the treatment of HIV has been studied for the treatment of COVID-19. In the TEMPEST trial which compared leronlimab to placebo in subjects with mild-to-moderate COVID-19, a *post hoc* analysis showed that leronlimab led to improvements from baseline in National Early Warning Score 2 (NEWS2) at Day 14 in the sub-set of people with more severe disease. Data has also been released on a further ongoing, randomized, placebo-controlled phase 3 registrational trial of leronlimab in 394 people with severe-to-critical COVID-19. The results show that Day 28 mortality was reduced (P ​= ​0.0319) in the subset of participants receiving leronlimab plus other pre-specified commonly used COVID-19 treatments including dexamethasone administered as part of their standard of care (SOC) compared to participants receiving placebo plus other pre-specified commonly used COVID-19 treatments including dexamethasone as part of their SOC. Several cases have recently been reported demonstrated that treatment with leronlimab restores immune function and achieves clinical improvement in people with critical COVID-19. Here we report on a further case of a critically ill person who was treated with leronlimab. This person had been on extracorporeal membrane oxygenation (ECMO) for an extended period of time before receiving 4 doses of leronlimab. The male subject received his first dose of leronlimab on Day 79 of hospitalization he was weaned off ECMO by Day 84 and discharged from the ECMO intensive care unit on Day 91.

## Highlights

• Leronlimab is a CCR5-specific monoclonal antibody. The CCR5 receptor is involved ​recruiting inflammatory cells.
• SARS-CoV-2 leads to a dysregulated host immune response which may mediate the morbidity and mortality of COVID-19.
• Leronlimab may disrupt CCR5-related recruitment of inflammatory cells, restoring immune function and improving survival.
• Cases of critically ill patients receiving leronlimab have been reported to be associated with recovery from COVID-19.

## List of abbreviations:

ARDS: acute respiratory distress syndrome
CCL3: chemokine C–C ligand 3 (or MIP-1α)
CCL4: chemokine C-C ligand 4
CCL5: chemokine C–C ligand 5 (or RANTES)
CCR5: C–C chemokine receptor type 5
COVID-19: coronavirus disease 2019
ECMO: extracorporeal membrane oxygenation
EIND: emergency investigational new drug
GWAS: genome-wide association study
IgG4: immunoglobulin G4
IL-6: interleukin-6
IL-10: interleukin-10
NEWS2: National Early Warning Score 2
RANTES: regulated on activation, normal T expressed and secreted (also known as CCL5)
RT–PCR: reverse transcriptase polymerase chain reaction
SARS-CoV-2: severe acute respiratory syndrome coronavirus 2
SOC: standard of care

## 1 Introduction

Although many people infected with SARS-CoV-2 remain asymptomatic or only suffer mild disease, in a substantial minority of people the disease can be severe or critical leading to hypoxic respiratory failure, shock, end-organ failure, and death [1,2]. Although mass vaccinations still remains the most promising approach to globally control the pandemic, disturbing evidence is emerging that new variants of the virus have been identified that may be able to evade the immune responses triggered by existing vaccines and natural immunity as a result of previous infections with the virus [3]. So while the recent approval of a number of SARS-CoV-2 vaccines provides hope there remains a considerable urgency for the availability of effective treatments for Coronavirus disease 2019 (COVID-19).

Data suggest that a key factor in the pathogenesis of severe COVID-19 are pro-inflammatory cytokines and chemokines from activated macrophages, initially in the lungs but then systemically [4,5]. Patients with severe-to-critical COVID-19 have been shown to have elevated levels of C–C chemokine receptor type 5 (CCR5) ligands including C–C ligand (CCL) 3 (or MIP-1α), CCL4 (or MIP-1β), CCL5 (or RANTES), as well as interleukin (IL) 6 and IL-10 [[6], [7], [8], [9], [10]].

Recently a genome-wide association study (GWAS) among people with severe COVID-19 has shown a strong association with genetic variants in the region on the short arm of chromosome 3 to which CCR5 maps [11]. There has also been an intriguing observation that people with the CCR5-Δ32 gene variant, which results in a non-functional CCR5 receptor, are less likely to suffer severe COVID-19 [12].

Considering the apparent role of the chemokine receptor-ligand system in the morbidity and mortality associated with COVID-19 pharmaceutical approaches that target this system have been proposed as a promising approach to treatment [13]. Indeed recent reports from clinical trials with IL-6 receptor antagonists, tocilizumab and sarilumab have suggested these agents may provide some benefit in subjects with critical COVID-19 [14,15]. However, it should be cautioned that other reports have failed to show a benefit from the IL-6 receptor antagonist tocilizumab, and even suggested that addition of tocilizumab to standard of care (SOC) may have been associated with increase mortality [16].

Another drug in development for COVID-19 is leronlimab, a C–C chemokine receptor type 5 (CCR5)-specific humanised IgG4 monoclonal antibody originally developed for the treatment of HIV for which it has been shown to be effective and well tolerated [[17], [18], [19]]. The recently completed TEMPEST trial (NCT04343651) compared leronlimab to placebo in subjects with mild-to-moderate COVID-19 [20]. Although the trial did not meet its primary efficacy endpoint, in a *post hoc* analysis in the subset of subjects with more severe disease a higher proportion of leronlimab-treated subjects (50%; 24/48) versus placebo-treated subjects (21%; 5/24) showed improvement in National Early Warning Score 2 (NEWS2) – a risk score for rapid clinical deterioration requiring critical care intervention (*post hoc* p ​= ​0.0223). Data has also been released on the CD12_COVID-19 trial (NCT04347239), which is an ongoing, randomized, placebo-controlled phase 3 registrational trial of leronlimab in 394 people with severe-to-critical COVID-19. The results show that Day 28 mortality was reduced (P ​= ​0.0319) in the subset of participants receiving leronlimab plus other pre-specified commonly used COVID-19 treatments including dexamethasone administered as part of their standard of care (SOC) compared to participants receiving placebo plus other pre-specified commonly used COVID-19 treatments including dexamethasone as part of their SOC [21]. Among all patients the difference in Day 28 mortality between leronlimab and placebo was not statistically different highlighting the importance of identifying and targeting treatments to specific subgroups of patients. Furthermore, a number of cases studies have recently been reported among subjects with critical COVID-19 showing leronlimab restored immune function and achieves clinical improvement as measured, for example, by vasopressor support, discontinuation of haemodialysis and mechanical ventilation [6,9,10].

## 2 Case description

The male subject was admitted to a London teaching hospital in the United Kingdom with confirmed nasopharyngeal swabs positive for SARS-CoV-2 infection by reverse transcriptase polymerase chain reaction (RT–PCR). He is of mixed race in his late 50’s with critical COVID-19. Other pertinent characteristics include a BMI of 37 kg/m^2^, prior smoker, and well-controlled hypertension. Following a positive test for SARS-CoV-2 infection, the subject was admitted to hospital with dyspnoea and pyrexia (Day 0). The subject received several investigational treatments prior to treatment with leronlimab. Upon admission, the subject was treated with dexamethasone for 10 days. Remdesivir was initiated on Day 1, and a plasma exchange was administered on Day 4 for 10 days. Other drug interventions included intravenous antibiotics. The subject’s condition continued to deteriorate, and extracorporeal membrane oxygenation (ECMO) was initiated on Day 19. Four doses of leronlimab (700 mg), obtained from CytoDyn (CytoDyn inc. WA, USA) through an Emergency Investigational New Drug (EIND) application, were administered on Days 79, 86, 93, and 100 post diagnosis. The subject responded extremely rapidly and was weaned off ECMO between Days 82–84, and he was discharged from the ECMO intensive care unit on Day 91. No adverse safety issues were identified with the administration of leronlimab in this subject. Oxygen therapy and intravenous antibiotics for ventilator-associated pneumonia were administered post weaning off ECMO.

## 3 Discussion

Recent data suggest that severely dysregulated host immune responses to SAR-CoV-2, referred to as ‘cytokine storm’, may predominately mediate the morbidity and mortality of severe-to-critical COVID-19 [13,22,23]. Cytokine storm primarily leads to lung inflammation causing acute respiratory distress syndrome (ARDS), but cytokine storm can also result in severe extrapulmonary manifestations, including thrombotic complications, myocardial dysfunction, and liver and kidney injury [24]. CCR5-expressing proinflammatory immune cells such as activated T-cells and macrophages are thought to play a key role in the cytokine storm response to COVID-19, suggesting that drug interventions that target the CCR5 system may represent a promising approach to treating COVID-19 [13].

Leronlimab is a C–C chemokine receptor type 5 (CCR5)-specific humanised IgG4 monoclonal antibody. In a Phase 2, placebo-controlled, randomized clinical trial in subjects with mild-to-moderate COVID-19 leronlimab was shown to provide clinical benefit, primarily in subjects with more severe disease. A number of recent case series have demonstrated that treatment with leronlimab restores immune function and achieves clinical improvement in people with critical COVID-19. Taken together therapeutic interventions that target the chemokine receptor–ligand system may be an effective approach to treating COVID-19 because chemotaxis and trafficking of CCR5 expressing proinflammatory immune cells are thought to play an important role in this process. Importantly the long-term safety of leronlimab is already well established in the treatment of HIV [[17], [18], [19]].

This case is of particular interest because to the best of our knowledge this subject received ECMO for the longest period of any person in the United Kingdom with COVID-19 (66 days). He received his first dose of leronlimab on Days 79 after diagnosis and was successfully weaned off ECMO between Days 82–84 and discharged from the ECMO intensive care unit on Day 91. Considering the length of time this subject was on ECMO and the speed of the subject’s response to leronlimab we believe that this case adds critical insight to the growing body of evidence for leronlimab in treatment of critical COVID-19.

## Credit roles

Dr. Elneil: Conceptualization; Data curation; Writing – review & editing, Dr. Lalezari: Writing – review & editing, Dr. Pourhassan: Resources; Writing – review & editing.

## Authors contributions

All authors attest that they meet the current International Committee of Medical Journal Editors (ICMJE) criteria for Authorship.

## Human and animal rights

The authors declare that the work described has not involved experimentation on humans or animals.

## Informed consent and patient details

The authors declare that informed consent was obtained. Treatment was conducted in accordance with the FDA requirements.

## Funding

This research did not receive any specific grant from funding agencies in the public, commercial, or not-for-profit sectors.

## Declaration of competing interest

The authors declare the following financial interests/personal relationships which may be considered as potential competing interests:

Sohier Elneil – nothing to declare.

Jacob Lalezari – is the investigator on CytoDyn's HIV and Oncology programs

Nader Pourhassan – is an employee of CytoDyn
